# Insights into Metabolically Healthy Obesity—A Narrative Review

**DOI:** 10.3390/jcm15166446

**Published:** 2026-08-20

**Authors:** Oana-Renada Roșca, Cristina Tudoran

**Affiliations:** 1 Department of General Medicine, Doctoral School, “Victor Babes” University of Medicine and Pharmacy, Eftimie Murgu Square, Nr. 2, 300041 Timisoara, Romania; oana.rosca@umft.ro; 2 Centre of Molecular Research in Nephrology and Vascular Disease, “Victor Babes” University of Medicine and Pharmacy, Eftimie Murgu Square, Nr. 2, 300041 Timisoara, Romania; 3 Department VII, Internal Medicine II, Discipline of Cardiology, “Victor Babes” University of Medicine and Pharmacy, Eftimie Murgu Square, Nr. 2, 300041 Timisoara, Romania; 4 County Emergency Hospital “Pius Brinzeu” Timisoara, L. Rebreanu, Nr. 156, 300723 Timisoara, Romania

**Keywords:** metabolically healthy obesity, cardiovascular risk, apolipoprotein B, visceral adiposity, cardiorespiratory fitness, sarcopenic obesity, dyslipidemia

## Abstract

**Background**: Obesity is a heterogeneous condition with variable cardiometabolic risk (CMR). The concept of metabolically healthy obesity (MHO) challenges the traditional paradigm that excess adiposity is invariably associated with metabolic dysfunction. However, its clinical validity remains controversial. **Methods**: A comprehensive literature search was conducted in PubMed, Scopus, Web of Science, and Google Scholar, primarily covering studies published between 2020 and 2025, with landmark earlier studies included when relevant. **Results**: Traditional definitions of MHO rely predominantly on metabolic parameters and fail to capture residual cardiovascular risk (CVR). Individuals with obesity frequently exhibit discordance between low-density lipoprotein cholesterol (LDL-C) and atherogenic particle burden, reflected by elevated apolipoprotein B and remnant lipoproteins. Visceral adiposity and reduced skeletal muscle mass further contribute to metabolic dysfunction. Cardiorespiratory fitness emerges as a major determinant of cardiometabolic resilience, independently associated with reduced mortality. **Conclusions**: Current evidence suggests that although some individuals may maintain metabolic health over extended periods, MHO frequently represents a dynamic phenotype that may progress toward metabolic dysfunction over time. An integrated approach incorporating lipid burden, body composition, and functional capacity enables more accurate CVR stratification and supports phenotype-based personalized strategies.

## 1. Introduction

In the 21st century, obesity has reached pandemic proportions and represents one of the most important preventable causes of cardiovascular disease (CVD) worldwide. According to recent global estimates, over one billion adults are currently living with obesity, with prevalence continuing to rise [1]. Beyond its epidemiological burden, obesity is increasingly recognized as a heterogeneous metabolic condition rather than a uniform CVR factor [2].

Although excess adiposity is traditionally associated with dyslipidemia, insulin resistance (IR), systemic inflammation, arterial hypertension, and coronary artery disease [3,4], emerging evidence suggests that a subset of individuals with obesity may remain relatively protected from these complications. This phenotype, termed MHO, is characterized by preserved insulin sensitivity, normal blood pressure, and a relatively favorable metabolic profile despite elevated body mass index (BMI) [2]. Cross-sectional studies estimate that 10–30% of individuals with obesity may fulfil criteria for MHO, depending on the diagnostic criteria applied [5,6,7].

Historically, no universally accepted definition of MHO has been established. Over the past two decades, several diagnostic criteria have been proposed, including those based on the Adult Treatment Panel III (ATP III) metabolic syndrome criteria, the Wildman definition, the Karelis Criteria, insulin sensitivity-based classifications, and more recently harmonized metabolic syndrome definitions. These approaches differ substantially with respect to the number and type of metabolic abnormalities permitted, the inclusion of inflammatory markers or IR, and the assessment of adiposity [4,6,8,9,10].

The earliest studies defined MHO using the Adult Treatment Panel III (ATP III-2001) [8] metabolic syndrome criteria. Individuals with obesity were considered metabolically healthy if they met fewer than three components of the metabolic syndrome. This pragmatic approach facilitated widespread use in epidemiological studies but evaluated only overt metabolic abnormalities, without considering IR, body fat distribution, or systemic inflammation.

Karelis et al. (Karelis Criteria 2004) [9] proposed a more restrictive definition focused on preserved metabolic function rather than the simple absence of metabolic syndrome. Their criteria incorporated a favorable lipid profile, insulin sensitivity, and low-grade inflammation, identifying a subgroup with lower CMR. However, several biomarkers are not routinely measured in clinical practice, limiting widespread implementation.

In 2008, Wildman [10] expanded the concept by integrating inflammatory markers and IR into the assessment of metabolic health. By allowing no more than one metabolic abnormality, this definition better captured the early stages of metabolic dysfunction. Nevertheless, differences in biomarker thresholds have contributed to considerable heterogeneity among studies.

More recent approaches recognize IR and visceral adiposity as central drivers of obesity-related risk. Accordingly, MHO has increasingly been classified using measures of insulin sensitivity (homeostatic model assessment for (HOMA-IR) or hyperinsulinemic clamp) together with imaging-derived assessment of visceral and ectopic fat by computed tomography (CT), magnetic resonance imaging (MRI), or dual-energy X-ray absorptiometry (DXA) [4,11]. These approaches provide a more pathophysiological characterization of obesity but remain limited by cost, availability, and lack of standardized cut-offs [12,13].

Overall, the evolution of MHO definitions reflects a transition from simple metabolic syndrome criteria toward multidimensional phenotyping integrating insulin sensitivity, fat distribution, inflammation, and advanced cardiometabolic biomarkers. Despite improved biological relevance, the absence of a universally accepted definition remains a major challenge for comparing studies and translating MHO into routine clinical practice [6].

However, the clinical validity of this phenotype remains controversial. Longitudinal data indicate that up to half of individuals with MHO transition to a metabolically unhealthy state within 5–10 years, suggesting that MHO may represent a transient condition rather than a stable phenotype [14]. Moreover, even in the absence of overt metabolic abnormalities, individuals with MHO exhibit higher cardiovascular event rates compared to metabolically healthy normal-weight individuals, indicating the presence of residual CVR not captured by conventional markers [15,16].

These observations suggest that conventional metabolic criteria alone may be insufficient to characterize CVR in obesity.

At the same time, other longitudinal studies have reported that a subset of individuals with MHO may preserve a favorable metabolic profile and experience relatively low CVR over prolonged follow-up [6,12]. These findings suggest that the clinical significance and long-term stability of MHO remain a matter of ongoing debate, emphasizing the need for a balanced evaluation of the available evidence [6,13].

Given the heterogeneity of existing diagnostic definitions, this review critically evaluates whether MHO represents a truly low-risk phenotype or a transient state of metabolic compensation.

Given the broad and multifactorial nature of MHO, this narrative review does not aim to comprehensively address every determinant of metabolic health in obesity. Instead, it deliberately focuses on three complementary domains: (1) body composition and fat distribution, (2) atherogenic lipid burden and advanced lipid biomarkers, and (3) cardiorespiratory fitness and skeletal muscle function.

These domains were selected because growing evidence suggests that they provide clinically relevant information beyond conventional metabolic syndrome criteria and may help explain part of the heterogeneity observed among individuals with obesity. Together, they integrate structural, metabolic, and functional aspects of obesity that are increasingly recognized as important determinants of CVR [2,17,18].

Other factors influencing obesity phenotypes, including age, sex, ethnicity, menopausal status, genetic predisposition, lifestyle factors, socioeconomic determinants, and comorbidities, are also discussed throughout the manuscript as important modifiers of metabolic health and CVR, although they are not the primary focus of this review.

Accordingly, the objective of this review was not to generate pooled quantitative estimates but to provide a comprehensive and balanced critical appraisal of the current evidence, identify knowledge gaps, and discuss their implications for CVR stratification in individuals with obesity.

## 2. Materials and Methods

### 2.1. Review Design

This review was designed as a narrative review aiming to critically evaluate the current evidence regarding MHO, with particular emphasis on body composition, atherogenic lipid burden, and cardiorespiratory fitness as complementary determinants of CVR.

### 2.2. Literature Search Strategy

A comprehensive literature search was conducted in PubMed, Scopus, Web of Science (Clarivate), and Google Scholar (last accessed on 10 August 2026). The search primarily focused on studies published between 2020 and 2025, while landmark publications published before 2020 were included when considered essential for understanding the evolution of MHO definitions and the pathophysiological mechanisms underlying obesity-related CVR. The search strategy combined keywords related to “metabolically healthy obesity”, “body composition”, “visceral adiposity”, “skeletal muscle”, “sarcopenic obesity”, “apolipoprotein B”, “lipoprotein(a)”, “remnant cholesterol”, “cardiorespiratory fitness”, “exercise capacity”, and “cardiovascular risk”. Boolean operators (AND/OR) were used to combine search terms, and database-specific indexing terms were applied whenever appropriate. The literature search was updated during manuscript revision to incorporate recently published evidence relevant to the scope of the review.

### 2.3. Eligibility Criteria

Studies were eligible for inclusion if they met the following criteria: (1) published in English; (2) full-text articles available for critical appraisal; (3) primarily published between 2020 and 2025, with landmark publications included when scientifically justified; (4) involving adult populations (≥18 years); and (5) addressing obesity phenotypes, metabolic health, body composition, lipid metabolism, physical fitness, or CVR.

Editorials, conference abstracts, letters to the editor, animal studies, in vitro studies, studies involving pregnant women, and non-peer-reviewed publications were excluded.

To enable comprehensive evaluation of the available evidence, studies with available full texts, in English, were preferentially included to enable critical appraisal of the original data. This approach may have resulted in the exclusion of some relevant publications that were not available in full-text format. This limitation is further addressed in Section Limitations.

### 2.4. Study Selection and Evidence Synthesis

Following duplicate removal using Zotero reference management software, titles and abstracts were independently screened by both authors according to the predefined eligibility criteria. Potentially relevant studies subsequently underwent full-text evaluation to determine their suitability for inclusion in this review.

Disagreements regarding study eligibility or interpretation of the available evidence were resolved through discussion until consensus was reached. Because this manuscript represents a narrative review, no quantitative synthesis or meta-analysis was performed. Instead, the evidence was critically synthesized by comparing findings across studies, identifying areas of consistency and disagreement, and considering potential methodological explanations for conflicting results. Greater weight was assigned to systematic reviews, meta-analyses, large prospective cohort studies, and contemporary international clinical guidelines, while smaller exploratory studies were interpreted within the context of the overall body of evidence. Other important factors taken into account were study size and the number of citations.

The primary objective of the evidence synthesis was not to quantify pooled effect estimates but to critically evaluate current knowledge regarding obesity phenotypes, identify consistent patterns across the literature, highlight existing controversies, and discuss their potential clinical implications.

### 2.5. Methodological Considerations

As this review represents a narrative review rather than a systematic review or meta-analysis, no formal risk-of-bias tool (e.g., the Newcastle–Ottawa Scale or ROBINS-I) was systematically applied across all included studies. However, narrative review articles included in the evidence synthesis were specifically appraised using the Scale for the Assessment of Narrative Review Articles (SANRA). The SANRA tool comprises six domains: justification of the article’s importance, statement of concrete aims or formulation of questions, description of the literature search, referencing, scientific reasoning, and appropriate presentation of data. Each domain was scored from 0 to 2, yielding a maximum total score of 12.

A total of 14 narrative reviews were assessed using the SANRA tool. Total SANRA scores ranged from 10/12 to 12/12, with a median score of 11/12. Of the 14 reviews evaluated, one (7.1%) achieved a score of 12/12, 12 (85.7%) scored 11/12, and one (7.1%) scored 10/12. Overall, the included narrative reviews demonstrated consistently high methodological quality according to the SANRA domains. The literature-search domain showed the greatest variability across the assessed reviews, reflecting differences in the level of detail with which search strategies were reported. Individual item scores are presented in Supplementary Table S1 [19].

**Figure 1:**
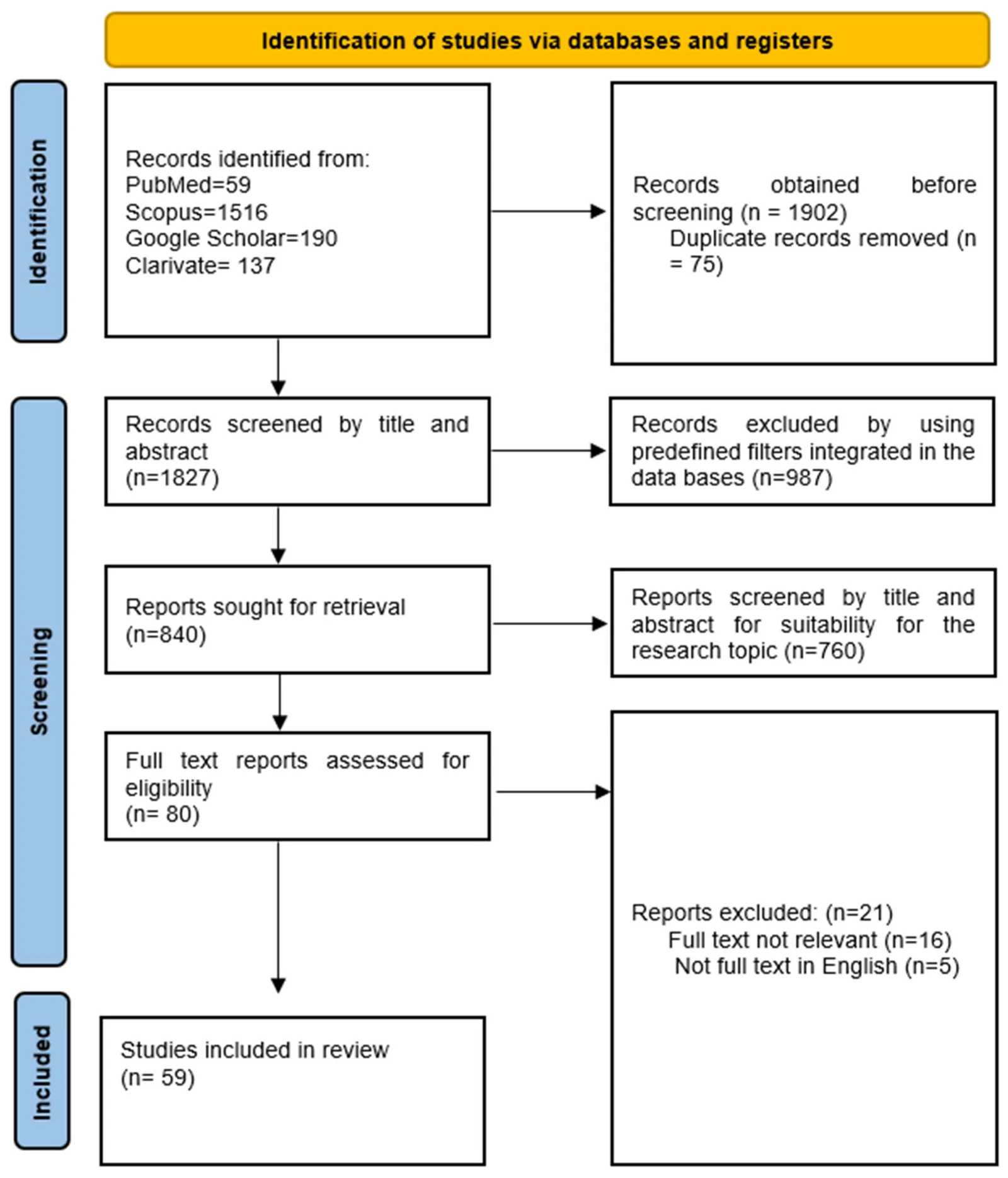
PRISMA 2020 flow diagram illustrating the literature search and study selection process performed for this narrative review.

The literature identification and study selection process is summarized in the Preferred Reporting Items for Systematic Reviews and Meta-Analyses (PRISMA 2020) flow diagram (Figure 1), which is presented to enhance transparency of the search strategy and study selection process, rather than to indicate that this review was conducted as a systematic review. After removing duplicates and applying the above-mentioned inclusion and exclusion criteria, a total of 59 manuscripts remained; see Figure 1.

## 3. Body Composition and CVR

The heterogeneity of CVR in obesity is largely driven by differences in body composition, particularly the distribution and function of adipose tissue [20,21,22,23,24]. Traditional anthropometric measures such as BMI fail to capture these nuances, as they do not differentiate between fat and lean mass, nor between metabolically distinct fat depots. A refined analysis of body composition therefore provides essential insights into why some individuals with obesity remain metabolically healthy, while others develop severe cardiometabolic complications [7,8,9].

### 3.1. Pathophysiological Pathways Linking Body Composition to Cardiovascular Disease

#### 3.1.1. Adipose Tissue as an Endocrine Organ

Adipose tissue functions as an endocrine and immune organ that secretes bioactive molecules known as adipokines, including leptin, adiponectin, resistin, and visfatin [25,26]. Dysregulation of adipokine secretion in obesity leads to IR, endothelial dysfunction, and systemic inflammation [27,28,29]. Adiponectin, typically reduced in visceral obesity, has anti-inflammatory and anti-atherogenic effects by enhancing nitric-oxide production and suppressing vascular adhesion molecules [30]. Conversely, elevated leptin levels stimulate sympathetic activation and vascular smooth-muscle proliferation, promoting arterial hypertension and atherogenesis [2,21,25,27].

#### 3.1.2. IR and Lipid Overflow

Visceral adipocytes exhibit enhanced lipolytic activity and resistance to insulin-mediated suppression of lipolysis, resulting in increased free fatty acids (FFAs) in the portal vein [5,14,31,32].

#### 3.1.3. Ectopic Fat and Organ Dysfunction

When adipose storage capacity is exceeded, lipids accumulate ectopically in the liver (non-alcoholic fatty liver disease (NAFLD)), heart (epicardial and myocardial fat), and skeletal muscle [25,33,34,35].

#### 3.1.4. Cardiorespiratory Fitness and Lean Mass as Modifiers

Cardiorespiratory fitness and muscle mass profoundly modify the metabolic consequences of obesity [36,37,38]. High levels of aerobic fitness attenuate IR, reduce visceral fat, and improve lipid oxidation [17,39,40]. Conversely, low fitness amplifies the impact of excess adiposity on inflammation and dyslipidemia.

### 3.2. Body Composition Parameters

#### 3.2.1. Traditional Anthropometric Indices

##### Body Mass Index

BMI remains a simple and universally applied tool for obesity classification [41,42]. However, it correlates only modestly with CVR because it does not account for fat distribution, age-related sarcopenia, or ethnic differences in body composition. Individuals with similar BMIs can exhibit widely divergent metabolic and lipid profiles, illustrating that “how fat is stored” matters more than “how much fat there is” [18,20,22,41]. Consequently, reliance on BMI alone can both underestimate risk in normal-weight individuals with visceral adiposity and overestimate it in physically active subjects with high muscle mass [43,44].

##### Waist Circumference (WC), Waist-to-Hip Ratio (WHR), and Waist-to-Height Ratio (WHtR)

Central obesity indices provide a better estimation of central fat distribution than BMI and are therefore more closely linked to CMR [41,45,46].

WC is a simple anthropometric measurement reflecting abdominal fat accumulation [26]. The World Health Organization recommends WC ≥ 94 cm in men and ≥80 cm in women as thresholds for increased metabolic risk, although ethnicity-specific cut-offs are increasingly recognized.

WHR calculated as WC divided by hip circumference captures fat distribution rather than absolute fat mass [25]. A higher WHR reflects central (android) fat predominance, whereas a lower ratio indicates greater gluteofemoral fat deposition, which has protective metabolic properties due to its lower lipolytic activity and greater insulin sensitivity. WHR has been consistently associated with cardiovascular morbidity and mortality independent of BMI.

WHtR, defined as WC divided by height, adjusts central adiposity for body size [41,42]. A WHtR ≥ 0.5 has been proposed as a simple and practical universal threshold for increased CMR across sexes and ethnicities. Compared with BMI, WHtR demonstrates improved predictive value for incident diabetes and CVD in several cohorts [2,3]. Collectively, these indices refine CVR stratification by capturing fat distribution, an essential determinant of metabolic dysfunction in obesity.

##### Hip Circumference (HC) and Gluteofemoral Adiposity

Although less commonly used as a standalone index, hip circumference plays an important role in interpreting WHR and assessing gluteofemoral fat depots, which play a metabolic protective role. Adipose tissue in this region displays low lipolytic activity, greater insulin sensitivity, and a high capacity for buffering circulating FFAs, thereby reducing ectopic lipid deposition in the liver, skeletal muscle, and pancreas. Large cohort studies have shown that higher HC is associated with reduced cardiovascular and all-cause mortality, even in individuals with elevated BMI.

##### Mid-Upper Arm Circumference (MUAC)

MUAC is primarily used to assess global nutritional status, especially in epidemiological and geriatric contexts. Among adults, MUAC serves as an indirect indicator of muscle mass and is associated with mortality risk in frail populations. In obesity, its value in determining CVR is limited; however, MUAC can aid in identifying sarcopenic obesity, a phenotype defined by excess adiposity in conjunction with reduced muscle mass.

##### Conicity Index and Sagittal Abdominal Diameter

The Conicity Index mathematically reflects how much an individual’s body shape deviates from a cylindrical shape, integrating weight, height, and WC to capture abdominal fat accumulation.

The Sagittal Abdominal Diameter directly measures anterior–posterior abdominal depth and correlates closely with visceral fat area, outperforming WC in some cohorts for predicting IR and subclinical atherosclerosis [41].

##### The Visceral Adipose Tissue/Subcutaneous Adipose Tissue (VAT/SAT) Ratio and Visceral Fat Mass (VFM)

Adipose tissue is not a uniform organ. Visceral adipose tissue (VAT), located around abdominal organs, is metabolically active, releasing free fatty acids (FFAs) and inflammatory mediators directly into the portal circulation. Subcutaneous adipose tissue (SAT), conversely, acts as a relatively inert lipid reservoir with higher insulin sensitivity and less pro-inflammatory activity. The VAT/SAT ratio, quantifiable by imaging modalities such as computed tomography (CT), magnetic resonance imaging (MRI), or dual-energy X-ray absorptiometry (DXA), is therefore a superior predictor of CMR risk compared with absolute fat mass [45].

VFM is a measure of the amount of adipose tissue surrounding the intra-abdominal organs and serves as an indicator of visceral adiposity [11,47]. Unlike subcutaneous fat, VFM is highly metabolically active and is strongly associated with IR, systemic inflammation, atherogenic dyslipidemia, and increased CVR [4,46,48]. It can be quantified using imaging techniques such as CT or MRI, as well as estimated by DXA and validated bioelectrical impedance analysis (BIA) devices [25,41,43,49].

Several studies suggest that the VAT/SAT ratio may represent a more accurate predictor of CVR than BMI alone, although its comparative performance varies across populations and imaging methodologies [6,12,46,49].

Several imaging-based cohort studies have demonstrated that visceral adiposity measures provide incremental predictive value beyond BMI alone [48,49]. In these studies, indices incorporating VAT showed stronger associations with incident cardiovascular events and cardiometabolic abnormalities than anthropometric measures alone [47,50]. However, the magnitude of this advantage varied according to imaging modality, ethnicity, sex, and population, highlighting the need for standardized assessment protocols [49,51].

##### Fat-Free Mass (FFM), Fat-Free Mass Index (FFMI) and FFM/FFMI Ratio

Beyond adiposity, FFM—comprising skeletal muscle, organs, and bone is an important determinant of metabolic health. This phenotype combines increased fat mass with decreased muscle mass or strength and is associated with markedly elevated cardiovascular and all-cause mortality.

The FFMI represents FFM normalized to height squared (kg/m^2^), analogously to BMI. By isolating the lean compartment from adiposity, FFMI provides a more physiologically meaningful assessment of body composition than BMI, which fails to distinguish between fat and lean tissues. FFMI can be derived from validated body composition techniques, primarily DXA and, in standardized settings, BIA. Height normalization enables interindividual comparisons and facilitates clinical interpretation across sexes and age groups. Proposed reference ranges vary by population, but low FFMI consistently reflects reduced lean tissue reserves and impaired metabolic and functional capacity [43].

From a clinical perspective, FFMI may complement conventional anthropometric indices by identifying reduced lean mass in individuals with similar BMI values [44]. However, despite its physiological relevance, FFMI remains primarily a research tool. The absence of universally accepted reference values, heterogeneous measurement techniques, and limited prospective validation currently preclude its routine use for CVR stratification in MHO.

The FFM/FFMI ratio has been proposed as an adjunctive research parameter for characterizing body composition. Its clinical utility remains uncertain because standardized cut-off values and prospective outcome validation are currently lacking.

Accordingly, the FFM/FFMI ratio should currently be regarded as an exploratory research parameter rather than a validated clinical tool for CVR assessment [44,52].

#### 3.2.2. Emerging Indices and Imaging Tools

These indices provide complementary information but are not routinely used in clinical decision-making [46,51].

Advances in body-composition imaging have enabled precise quantification of regional adiposity. Techniques such as MRI and CT accurately assess visceral, subcutaneous, and ectopic fat depots, while DXA provides reproducible estimates of total and regional fat and lean mass. BIA, although less precise, is suitable for large-scale or clinical settings. Emerging composite indices—such as the visceral adiposity index (VAI) and body adiposity index (BAI)—integrate anthropometric and lipid parameters, offering additional risk discrimination [46,48,51].

##### Visceral Adiposity Index (VAI)

The Visceral Adiposity Index (VAI) is a sex-specific composite index. Higher VAI values are associated with IR, NAFLD, and an atherogenic lipid profile, even in individuals with normal BMI. VAI therefore bridges the gap between simple anthropometry and advanced imaging, offering a practical tool for identifying high-risk obesity phenotypes in routine clinical settings.

However, its routine clinical use remains limited by the lack of universally accepted cut-off values and prospective validation [46,48,51].

##### Body Adiposity Index (BAI)

The BAI, calculated from hip circumference and height, was proposed as an alternative to BMI for estimating body fat percentage, particularly in populations where muscle mass and bone structure may distort BMI. Although BAI correlates reasonably with measured body fat, it does not capture fat distribution and appears less predictive of cardiometabolic outcomes than indices that reflect visceral adiposity (e.g., VAI or VAT/SAT ratio). BAI should currently be regarded as a complementary research tool rather than a replacement for BMI or WC in CVR assessment [41].

##### Lipid Accumulation Product (LAP)

The LAP is a composite index designed to quantify lipid overaccumulation in relation to central adiposity, thereby capturing metabolic risk more accurately than isolated anthropometric or lipid parameters. LAP combines WC and fasting triglyceride (TG) concentrations to estimate visceral lipid overaccumulation. Compared with anthropometric indices alone, LAP has shown good performance for identifying IR, metabolic syndrome, and CMR.

Conceptually, LAP reflects the pathophysiological interaction between visceral fat deposition and dyslipidemia, two core features of IR and cardiometabolic dysfunction. Elevated LAP values are strongly associated with hepatic steatosis, impaired glucose metabolism, systemic inflammation, and endothelial dysfunction, positioning LAP as a surrogate marker of ectopic lipid burden rather than total adiposity. In multiple populations, LAP has demonstrated superior predictive value for type 2 diabetes, metabolic syndrome, and atherosclerotic cardiovascular disease compared with BMI and WC alone. Nevertheless, its clinical interpretation is influenced by sex, ethnicity, and TG variability, and standardized cut-off values for risk stratification remain incompletely defined [53].

##### Body Shape Index (ABSI)

ABSI is an anthropometric indicator developed to quantify central adiposity independently of overall body size, thereby overcoming a key limitation of BMI. ABSI integrates WC, height, and weight using an allometric scaling approach.

By normalizing WC to body size, ABSI isolates abdominal fat distribution rather than absolute adiposity. Elevated ABSI values have been consistently associated with increased all-cause and cardiovascular mortality, reflecting the pathogenic role of visceral fat accumulation and its links to IR, systemic inflammation, and endothelial dysfunction. Unlike BMI, ABSI demonstrates a near-linear relationship with mortality risk across populations. However, its clinical applicability is limited by modest associations with metabolic biomarkers and reduced sensitivity for tracking changes in body composition over time.

##### Body Roundness Index (BRI)

The BRI is a geometric anthropometric index designed to estimate body fat distribution and visceral adiposity based on the relationship between WC and height. BRI is derived from a mathematical model, which approximates body shape to an ellipse.

BRI correlates strongly with total body fat percentage, VFM, and CMR factors, including dyslipidemia, IR, and arterial hypertension. Compared with BMI and WC alone, BRI provides improved discrimination of obesity-related metabolic risk and has shown predictive value for type 2 diabetes mellitus and CVD. Nevertheless, BRI remains an indirect estimate of adiposity, although it lacks universally accepted cut-off values, and may be influenced by ethnic and sex-specific body shape differences [41].

## 4. Lipid Profile in Obesity: Identifying the Most Dangerous Pattern

Obesity profoundly alters lipid metabolism, giving rise to a spectrum of dyslipidemia patterns that vary according to adipose distribution, insulin sensitivity, and hepatic lipid handling. The characteristic lipid profile of obesity (elevated TG, reduced HDL-C, and normal or modestly elevated LDL-C is often deceptively benign when assessed by conventional lipid panels. However, advanced lipid and apolipoprotein analyses reveal a more atherogenic milieu, dominated by small dense LDL (sd-LDL) particles, remnant lipoproteins, and increased ApoB concentrations.

This section outlines the pathophysiological mechanisms underlying these abnormalities and identifies the lipid phenotypes most predictive of CVD in obesity [30,51,54].

### 4.1. Pathophysiology of Obesity-Related Dyslipidemia

#### 4.1.1. IR and Hepatic Lipid Overproduction

The central driver of dyslipidemia in obesity is IR, a hallmark of VA. In the insulin-resistant state, adipose tissue fails to suppress lipolysis, leading to increased flow of FFAs to the liver via the portal vein. Hepatocytes respond by upregulating de novo lipogenesis and very low-density lipoproteins (VLDL) synthesis. Elevated hepatic TG production results in over-secretion of large, TG-rich VLDL1 particles. These particles undergo intravascular lipolysis mediated by lipoprotein lipase (LPL), yielding smaller remnant particles that are highly atherogenic [26,54].

Simultaneously, IR impairs LDL receptor activity and HDL-mediated reverse cholesterol transport, compounding lipid accumulation in the circulation and arterial wall [14,21,31,32,48].

#### 4.1.2. Adipose Tissue Inflammation and Adipokine Dysregulation

Inflamed adipose tissue releases cytokines such as TNF-α and IL-6, which further inhibit LPL and alter apolipoprotein (Apo) production [27,28]. Tumour necrosis factor α (TNF-α) suppresses hepatic ApoA-I and ApoA-II synthesis and accelerates HDL particle catabolism, thereby reducing HDL concentration and impairing reverse cholesterol transport. Meanwhile, interleukin-6 (IL-6) enhances hepatic synthesis of ApoB-containing lipoproteins and C-reactive protein (CRP), establishing a pro-inflammatory and pro-atherogenic environment [27].

Reduced adiponectin, a key anti-inflammatory adipokine, diminishes fatty acid oxidation and promotes hepatic steatosis, both of which exacerbate dyslipidemia [28,55].

#### 4.1.3. The Role of Ectopic Fat

Ectopic lipid deposition, particularly in the liver and visceral depots, amplifies lipid derangements. NAFLD, a common comorbidity in obesity, further promotes VLDL overproduction, while hepatic IR prevents suppression of lipogenesis.

In advanced NAFLD, secretion of ApoC-III, a potent inhibitor of LPL and hepatic remnant clearance, increases, leading to accumulation of TG-rich remnants that directly damage the vascular endothelium [25,33,34,35,56].

### 4.2. Common Lipid Patterns in Obesity

#### 4.2.1. Elevated TG and Reduced HDL-C

The combination of high TG and low HDL-C represents the core phenotype of obesity-related dyslipidemia [5,57,58]. This pattern is particularly prevalent in individuals with visceral obesity and IR. Elevated VLDL-TG promotes the exchange of lipids between VLDL and HDL via cholesteryl ester transfer protein (CETP), resulting in TG-enriched HDL particles that are rapidly catabolized [5].

The consequent reduction in HDL-C impairs reverse cholesterol transport and removes a key anti-inflammatory, antioxidant defence mechanism against atherogenesis [30].

Beyond conventional lipid parameters, TG/HDL-c ratio has emerged as a practical surrogate marker of insulin resistance and residual CMR, particularly in individuals with obesity and metabolic syndrome [5,57,58].

#### 4.2.2. Small Dense LDL (sd-LDL): The Silent Threat

Although total LDL-C levels in obesity may appear normal or only mildly elevated, qualitative alterations in LDL particles confer substantial CVR. Hypertriglyceridemia promotes the formation of sd-LDL particles through the action of cholesteryl ester transfer protein (CETP), a plasma glycoprotein that mediates TG–cholesterol exchange between HDL and ApoB-containing lipoproteins, followed by hepatic lipase–driven lipolysis [50].

Oxidized LDL (oxLDL) represents a biologically “activated” LDL fraction that amplifies vascular inflammation and plaque instability through scavenger receptor–mediated uptake (e.g., LOX-1) and downstream VAT endothelial dysfunction. In obesity and IR, increased oxidative stress and postprandial remnant accumulation enhance LDL susceptibility to oxidation, and circulating oxLDL has been consistently associated with subclinical atherosclerosis and incident atherosclerotic CVD beyond LDL-C alone [22]. Where available, oxLDL or related oxidized phospholipid assays may therefore refine residual risk stratification in patients with atherogenic dyslipidemia.

Sd-LDL particles are more atherogenic due to: (a) greater arterial wall penetration; (b) higher susceptibility to oxidation; (c) lower affinity for LDL receptors; (d) prolonged plasma half-life [50,51,54].

Sd-LDL particles are strongly associated with residual CVR. However, their measurement remains largely confined to specialized laboratories and research settings. Consequently, current clinical practice continues to rely primarily on ApoB and non-HDL cholesterol for routine assessment of atherogenic particle burden, while sd-LDL may provide complementary information in selected patients [47,50,54].

#### 4.2.3. ApoB and Non-HDL Cholesterol

ApoB quantifies the total number of circulating atherogenic lipoprotein particles (VLDL, IDL, LDL, and lipoprotein a—Lp(a)), as each particle contains a single ApoB molecule, providing a direct measure of atherogenic burden [50,54].

Obesity, especially the visceral phenotype, is associated with elevated ApoB and non-HDL-C, both superior predictors of CVD events compared to LDL-C. ApoB thus identifies patients with discordantly high particle numbers despite “normal” LDL-C, a frequent status in obesity and metabolic syndrome [30,50,51].

The TG/HDL-C ratio, an easily calculated surrogate marker, correlates strongly with IR and sd-LDL levels. A ratio > 3 mg/dL is often considered indicative of an atherogenic phenotype [5,57,58].

### 4.3. Advanced Lipid Biomarkers and Future Directions

#### 4.3.1. Lipoprotein(a) [Lp(a)]

While circulating Lp(a) concentrations are largely determined by LPA kringle IV type 2 repeat polymorphisms governing Lp(a) isoform size, obesity-related inflammation and metabolic dysregulation markedly amplify its pathogenic potential through oxidative and pro-inflammatory modifications [16]. In insulin-resistant states, these alterations enhance vascular retention and thrombogenicity, thereby accelerating atherothrombosis and valvular calcification [11,47].

**Figure 2:**
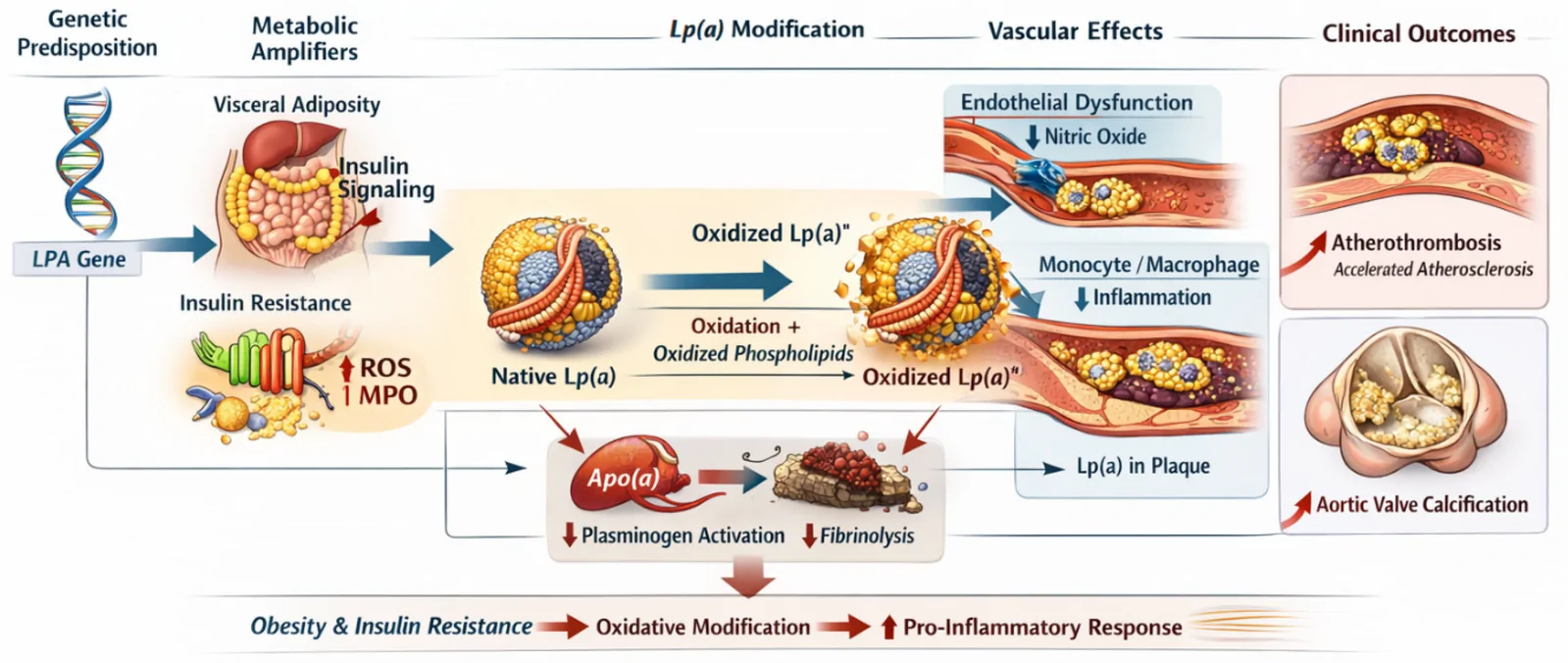
Metabolic amplification of lipoprotein(a)-mediated atherothrombosis and aortic valve calcification in obesity and IR. Legend: Apo(a) = apolipoprotein(a); Lp(a) = lipoprotein(a); LPA = lipoprotein(a) genes; ROS = reactive oxygen species; MPO = myeloperoxidase.

Inflammation represents a central amplifier for Lp(a)-related vascular injury, particularly in insulin-resistant states. Thus, although Lp(a) levels per se may remain stable, obesity and IR transform Lp(a) into a more biologically aggressive particle, linking metabolic dysfunction to accelerated atherothrombosis and calcific valvular disease [16], see Figure 2.

Current evidence does not support its use as a primary biomarker for distinguishing MHO from metabolically unhealthy obesity (MUO). In contrast, biomarkers reflecting adipose tissue dysfunction, such as ApoB, remnant cholesterol, and measures of visceral adiposity, appear more closely linked to obesity-related metabolic heterogeneity [24,48,50,51].

#### 4.3.2. Remnant Cholesterol

Remnant cholesterol, representing the cholesterol content of TG-rich lipoproteins (VLDL and IDL remnants), is emerging as a causal risk factor for atherosclerosis. It contributes directly to cholesterol deposition into the arterial wall and inflammation [54,59]. Patients with obesity often exhibit elevated remnant cholesterol despite normal LDL-C. Although growing evidence supports its contribution to residual CVR, routine assessment has not yet been universally adopted and current guidelines continue to prioritize ApoB and non-HDL cholesterol in clinical practice [50,51,54,59].

#### 4.3.3. Lipidomics and Metabolomic Profiling

Recent lipidomic studies reveal distinctive metabolic fingerprints in obesity, with elevated ceramides, diacylglycerols, and acylcarnitines promoting IR and endothelial dysfunction. These molecules represent both biomarkers and potential therapeutic targets for precision lipid management [20,54].

This constellation, consisting of high TG, low HDL-C, elevated ApoB, and predominance of sd-LDL, represents the lipid phenotype with the highest CVR in obesity. It is particularly associated with visceral adiposity, NAFLD, and low physical fitness [22,23], see Table 1.

### 4.4. Clinical Implications

Early recognition of this lipid pattern is essential for personalized prevention. Standard lipid panels should be complemented by:

- ApoB and non-HDL-C measurement for particle quantification
- TG/HDL-C ratio as an accessible screening marker [5]
- Imaging for visceral and hepatic fat to contextualize lipid abnormalities

Therapeutic strategies should address both lipid metabolism and underlying IR through weight reduction, physical activity, and pharmacological agents such as glucagon-like peptide-1 (GLP-1) receptor agonists, sodium-glucose cotransporter-2 inhibitors (SGLT2i), and statins and/or proprotein convertase subtilisin/kexin type 9 inhibitors (PCSK9i) when indicated [12,13].

## 5. Physical Activity, Skeletal Muscle Mass, and Cardiometabolic Protection

The metabolic heterogeneity of obesity extends beyond adipose tissue; skeletal muscle mass and function play an important and complementary role in determining the CVR of an individual [52]. Far from being a passive structure, skeletal muscle is metabolically active and an endocrine organ whose secretory profile, particularly the release of myokines, regulates lipid metabolism, insulin sensitivity, inflammation, and vascular function [44,51,52,61].

In the context of obesity, the degree of cardiorespiratory fitness and muscle quality can profoundly modify the impact of excess adiposity on lipid profile and CVR. These observations have contributed to the recognition that some individuals with obesity may temporarily preserve a favorable metabolic profile despite excess adiposity. Nevertheless, cardiorespiratory fitness should be regarded as one component of the MHO phenotype rather than its defining characteristic, since metabolically healthy individuals are not uniformly highly fit and highly fit individuals do not invariably fulfill accepted MHO criteria [7,51,52,61].

Rather than serving as a diagnostic criterion, cardiorespiratory fitness should be viewed as a functional modifier that influences CMR across obesity phenotypes [20,22].

### 5.1. Skeletal Muscle as a Metabolic Organ

#### 5.1.1. Insulin-Mediated Glucose and Lipid Uptake

Skeletal muscle is responsible for approximately 70–80% of postprandial glucose disposal under insulin stimulation. Muscle fibres also oxidize FFAs, thereby preventing lipid spillover into ectopic depots such as the liver and myocardium.

In obesity, chronic physical inactivity and intramuscular lipid accumulation impair insulin signalling pathways, notably via serine phosphorylation of insulin receptor substrate (IRS-1) and activation of protein kinase C. This leads to reduced glucose transporter type 4 (GLUT4) translocation to the sarcolemma, impaired glucose uptake, and lipid-induced IR [17].

Conversely, exercise training, particularly structured aerobic training and high-intensity interval training (HIIT), upregulates peroxisome proliferator-activated receptor gamma coactivator 1-alpha (PGC-1α)–driven mitochondrial biogenesis, increases GLUT4 expression and insulin-stimulated glucose transport, enhances skeletal muscle lipoprotein lipase activity, and improves intramyocellular lipid handling through increased β-oxidation and mitochondrial quality control. These adaptations restore metabolic flexibility and improve whole-body lipid homeostasis [39,40].

Collectively, preservation of skeletal muscle insulin sensitivity may contribute to maintaining the MHO phenotype by delaying the progression toward metabolic dysfunction and ectopic fat accumulation [20,44].

#### 5.1.2. Myokines and Endocrine Cross-Talk

Contracting muscles secrete myokines, bioactive peptides that exert endocrine effects on adipose tissue, liver, and the CV system. Among the best-studied are:

- Irisin—stimulates browning of white adipose tissue and increases energy expenditure; inversely correlated with visceral fat accumulation.
- IL-6 (exercise-induced form)—unlike its chronic inflammatory counterpart, transient IL-6 release during exercise enhances lipolysis and glucose uptake.
- Myonectin and fibroblast growth factor 21 (FGF21) improve hepatic lipid oxidation and reduce VLDL-TG synthesis.

Thus, skeletal muscle acts as a counter-regulatory system opposing adipose-derived pro-inflammatory cytokines [27,28].

These exercise-induced endocrine adaptations may partly explain the lower inflammatory burden observed in some individuals with MHO, although longitudinal studies are still needed to establish causality [20,38,44].

### 5.2. Cardiorespiratory Fitness

#### 5.2.1. Fitness as a Modifier of CVR

The concept of “MHO” is partially explained by differences in cardiorespiratory fitness (CRF) [20,44]. Individuals with high CRF exhibit lower visceral fat, improved insulin sensitivity, and a more favorable lipid profile compared to unfit individuals with similar BMI [37].

Prospective studies consistently demonstrate that each 1-MET increase in cardiorespiratory fitness is associated with an approximately 10–15% reduction in cardiovascular mortality. Most of these data originate from heterogeneous cardiovascular cohorts rather than exclusively obese populations. Available obesity-specific studies suggest a similar protective association, although its magnitude may vary according to age, sex, baseline metabolic status, and the criteria used to define MHO [38,51].

CRF reflects the integrative capacity of the cardiovascular, respiratory, and skeletal muscle systems to deliver and utilize oxygen during sustained physical activity. Higher CRF is associated with enhanced mitochondrial density, improved endothelial function, reduced visceral adiposity, and superior lipid handling, independently of BMI. These adaptations translate into lower ApoB concentrations, reduced TG-rich lipoproteins, and improved HDL functionality, providing a robust cardioprotective effect even in the presence of obesity [44,61].

#### 5.2.2. Mechanistic Links

Regular physical activity induces a broad spectrum of metabolic and vascular adaptations that collectively attenuate obesity-related CVR. Exercise increases LPL activity in skeletal muscle, thereby enhancing TG hydrolysis and accelerating clearance of TG-rich lipoproteins. This process reduces circulating residual particles and limits postprandial lipemia [61].

Concomitantly, physical training improves HDL metabolism by increasing HDL-C concentration and enhancing HDL functionality, particularly reverse cholesterol transport and antioxidant capacity. Exercise also suppresses hepatic VLDL secretion through improved insulin sensitivity and reduced FFA flow to the liver.

At the vascular level, regular exercise reduces systemic inflammation and oxidative stress by improving endothelial nitric oxide bioavailability, decreasing sympathetic overactivity, and modulating cytokine production. These integrated adaptations mitigate the atherogenic lipid triad typical of visceral obesity and contribute to long-term cardiovascular protection [62].

### 5.3. Sarcopenic Obesity: The Dual Risk

While high fitness confers substantial metabolic protection, the opposite phenotype—sarcopenic obesity—represents one of the highest-risk states for obesity-related CVD. This condition, characterized by excess fat mass combined with reduced skeletal muscle mass or strength, frequently develops in the context of aging, chronic inflammation, hormonal imbalance, and prolonged physical inactivity.

Sarcopenic obesity exacerbates IR through multiple mechanisms, including reduced mitochondrial oxidative capacity, impaired fatty acid uptake and oxidation, increased ectopic fat deposition, and decreased secretion of protective myokines such as irisin and IL-15. These alterations promote metabolic inflexibility and amplify lipid abnormalities [44].

Clinically, individuals with sarcopenic obesity display higher concentrations of ApoB, TG, and remnant cholesterol, together with impaired HDL functionality. Epidemiological studies consistently demonstrate that their cardiovascular and all-cause mortality risk exceeds that of individuals with obesity with preserved muscle mass, highlighting the synergistic pathogenic effects of adiposity and muscle loss [52].

### 5.4. Exercise Modalities and Lipid Modulation

#### 5.4.1. Aerobic Training

Endurance exercise constitutes the cornerstone of dyslipidemia management in obesity. Regular aerobic activity (≥150 min per week of moderate intensity) reduces TG levels by 10–30%, increases HDL-C by 5–10%, and decreases the proportion of sd-LDL particles. These effects are mediated by enhanced skeletal muscle LPL activity, improved hepatic lipid turnover, and reduced visceral fat mass [61].

Importantly, aerobic training preferentially reduces visceral and hepatic adiposity, even in the absence of significant weight loss, thereby directly targeting the most atherogenic fat depots [39].

#### 5.4.2. Resistance Training

Resistance exercise promotes muscle hypertrophy, improves insulin sensitivity, and increases basal metabolic rate, thereby facilitating long-term lipid oxidation. When combined with aerobic training, resistance exercise yields superior benefits on body composition and lipid profile, particularly through reductions in the TG/HDL-C ratio and ApoB concentration [17].

Emerging evidence indicates that resistance training also stimulates the secretion of metabolically beneficial myokines, including irisin and IL-7, contributing to systemic anti-inflammatory and lipid-modulating effects.

#### 5.4.3. High-Intensity Interval Training (HIIT)

HIIT provides time-efficient metabolic benefits through short bursts of intense effort interspersed with recovery periods. This training modality enhances mitochondrial density, insulin sensitivity, and lipid oxidation more effectively than continuous moderate exercise [40].

Intervention studies have demonstrated significant reductions in visceral adiposity and TG levels within weeks of HIIT initiation, positioning it as a practical strategy for high-risk individuals with obesity with limited time for prolonged training sessions [62].

### 5.5. Integration of Muscle and Fat Metrics in Risk Assessment

Traditional CVR models rarely incorporate indices of muscle mass or physical fitness, despite strong evidence supporting their prognostic relevance. Low FFMI and poor CRF are consistently associated with higher ApoB/ApoA1 and TG/HDL-C ratios, even among individuals with similar BMI values. This observation underscores that body composition quality, rather than total body mass, represents a key determinant of cardiometabolic health [43].

The combined assessment of visceral adiposity and skeletal muscle mass provides a substantially more accurate estimation of CVR than BMI alone. Integration of WC or imaging-derived fat measures with DXA- or bioimpedance-derived muscle indices enables identification of high-risk phenotypes such as sarcopenic obesity and low-fitness obesity, which often remain undetected by conventional anthropometry [41].

Furthermore, incorporation of functional parameters, including handgrip strength and CRF, adds a dynamic dimension to risk profiling by capturing physiological reserves and metabolic resilience. Consequently, multidimensional evaluation of fat distribution, muscle integrity, and physical performance should be regarded as a cornerstone of modern obesity-related CVR stratification.

#### Rationale for a Refined Risk Stratification

Traditional CVR models rely heavily on BMI as the main obesity measure, which has several limitations (it does not distinguish between fat and lean mass and it does not determine the regional distribution of adipose tissue). Consequently, a multidimensional approach integrating anthropometric, compositional, and biochemical parameters is required for accurate individual risk assessment.

Recent advances in imaging (DXA, CT, MRI) and bioimpedance technology enable precise quantification of visceral and subcutaneous fat depots, while biochemical markers such as ApoB, non-HDL-C, and remnant cholesterol offer deeper insight into lipid-driven atherogenesis. Furthermore, evaluating CRF and muscle mass is critical, as increased lean mass and high aerobic capacity mitigate many of the adverse effects of obesity on lipid metabolism and vascular health.

### 5.6. Indices of Exercise Capacity and Physical Fitness

Beyond body composition, objective assessment of exercise tolerance and physical fitness provides critical insight into cardiometabolic resilience in obesity. Several validated indices quantify cardiorespiratory capacity, functional reserve, and muscular endurance, offering complementary prognostic information.

- Peak Oxygen Uptake (VO_2_peak/VO_2_max)

Peak oxygen uptake represents the maximal capacity of the cardiovascular and muscular systems to transport and use oxygen during incremental exercise testing. Expressed in mL·kg^−1^·min^−1^, VO_2_peak is considered the gold standard for evaluating cardiorespiratory fitness. Higher VO_2_peak values are independently associated with reduced cardiovascular and all-cause mortality, improved lipid metabolism, and enhanced insulin sensitivity, regardless of BMI. In obesity, low VO_2_peak identifies individuals at particularly high CMR, even in the absence of overt metabolic abnormalities [37].

B. Metabolic Equivalent of Task (METs)

Exercise capacity can also be expressed in METs, where 1 MET corresponds to resting oxygen consumption (~3.5 mL O_2_·kg^−1^·min^−1^). Peak METs achieved during treadmill or cycle ergometry testing strongly predict cardiovascular outcomes. Each 1-MET increase is associated with a 10–15% reduction in mortality risk [36]. MET-based assessment is widely applicable in clinical settings due to its simplicity and strong prognostic value.

C. Ventilatory Threshold (VT) and Anaerobic Threshold (AT)

The ventilatory and anaerobic thresholds reflect the transition from aerobic to partially anaerobic metabolism during exercise. These parameters indicate submaximal endurance capacity and metabolic efficiency. In individuals with obesity, reduced VT/AT is associated with impaired mitochondrial function, early fatigue, and unfavorable lipid profiles. VT and AT are particularly useful for tailoring individualized exercise prescriptions.

D. Six-Minute Walk Test (6MWT)

The six-minute walk distance provides a practical, submaximal measure of functional exercise capacity. It reflects integrated cardiopulmonary, muscular, and motivational performance. In obesity, reduced walking distance correlates with higher visceral fat, lower FFMI, elevated ApoB, and increased CVR, making it a valuable screening tool in outpatient settings [38].

E. Handgrip Strength and Muscular Endurance Indices

Handgrip strength serves as a surrogate marker of global muscle function and physical resilience. Low grip strength is associated with adverse lipid profiles, IR, and increased mortality in obese populations. When combined with FFMI, it enables the identification of sarcopenic and functionally impaired phenotypes [44].

F. Supportive Assessment Tools

Validated instruments such as the International Physical Activity Questionnaire (IPAQ) or Global Physical Activity Questionnaire (GPAQ) provide supportive estimates of habitual activity levels. Although subjective, they complement objective fitness measures and aid longitudinal monitoring.

#### Clinical Integration

The combined assessment of VO_2_peak (or METs), submaximal endurance (VT/AT, 6MWT), and muscular strength enables comprehensive characterization of functional capacity in obesity. Individuals with preserved fitness demonstrated lower TG, improved HDL functionality, and reduced ApoB concentrations, even in the presence of excess adiposity [36,37,38]. Conversely, low exercise capacity identifies high-risk phenotypes that may not be captured by anthropometric or lipid indices alone [36,37,44].

Although multiple indices of physical fitness have been proposed, their clinical value is not equivalent among available measures. Among available measures, cardiorespiratory fitness assessed by peak oxygen uptake (VO_2_peak) during cardiopulmonary exercise testing has consistently demonstrated the strongest association with cardiovascular and all-cause mortality, independent of BMI and traditional CMR factors. Conversely, field tests such as the 6 min walk test primarily reflect functional capacity and are influenced by age, musculoskeletal limitations, and motivation, making them less specific for CMR stratification. Muscle strength assessments, particularly handgrip strength, provide complementary information regarding sarcopenia and frailty but are weaker predictors of cardiovascular outcomes when considered in isolation. Therefore, although each assessment contributes different clinical information, objective measures of cardiorespiratory fitness currently provide the most robust prognostic value in individuals with obesity [20,37,61].

Importantly, the predictive performance of these measures also differs according to clinical setting. CPET remains the reference standard because it directly quantifies aerobic capacity, but its routine implementation is limited by cost, equipment, and specialized expertise. Consequently, simpler surrogate measures such as the 6 min walk test or handgrip strength may be more feasible for large-scale clinical screening, although they sacrifice prognostic precision. Future studies should determine whether combining fitness measures with body composition and lipid biomarkers provides incremental value over individual assessments alone [51].

Based on the data extracted from the medical literature, we determined the main differences in body composition, metabolic profile and physical activity between high-fitness obesity MHO and MUHO.

Although obesity phenotypes are often described as distinct entities, current evidence suggests that they represent a continuum rather than clearly separated categories. High cardiorespiratory fitness, preserved metabolic health, and favorable body composition frequently coexist, whereas progressive visceral adiposity, IR, inflammation, and lipid abnormalities gradually characterize MUO. Accordingly, no single parameter is sufficient to distinguish these phenotypes; instead, CVR increases progressively as adverse metabolic and functional abnormalities accumulate.

Individuals with a more favorable phenotype generally present lower visceral adiposity, reduced ectopic fat accumulation, preserved skeletal muscle mass, greater cardiorespiratory fitness, better insulin sensitivity, lower ApoB concentrations, lower remnant cholesterol and TG levels, higher HDL-C, and reduced systemic inflammation. In contrast, progression toward MUO is accompanied by increasing visceral fat deposition, worsening IR, higher concentrations of ApoB, remnant lipoproteins and sd-LDL particles, declining cardiorespiratory fitness and muscle mass, and a progressively more pro-inflammatory profile. These alterations rarely occur simultaneously but accumulate gradually over time, reflecting the dynamic nature of obesity phenotypes.

Taken together, these observations support replacing rigid categorical classifications with a multidimensional assessment integrating adipose tissue distribution, lipid abnormalities, metabolic function, and physical fitness. Such an approach better reflects the biological complexity of obesity and may improve cardiovascular risk stratification beyond BMI alone.

However, MHO is often transient: longitudinal studies reveal that nearly half of MHO individuals develop metabolic syndrome within 5–10 years, particularly if physical activity declines [6,48]. Thus, the MHO phenotype represents a dynamic and modifiable state, not an inherently benign one.

**Figure 3:**
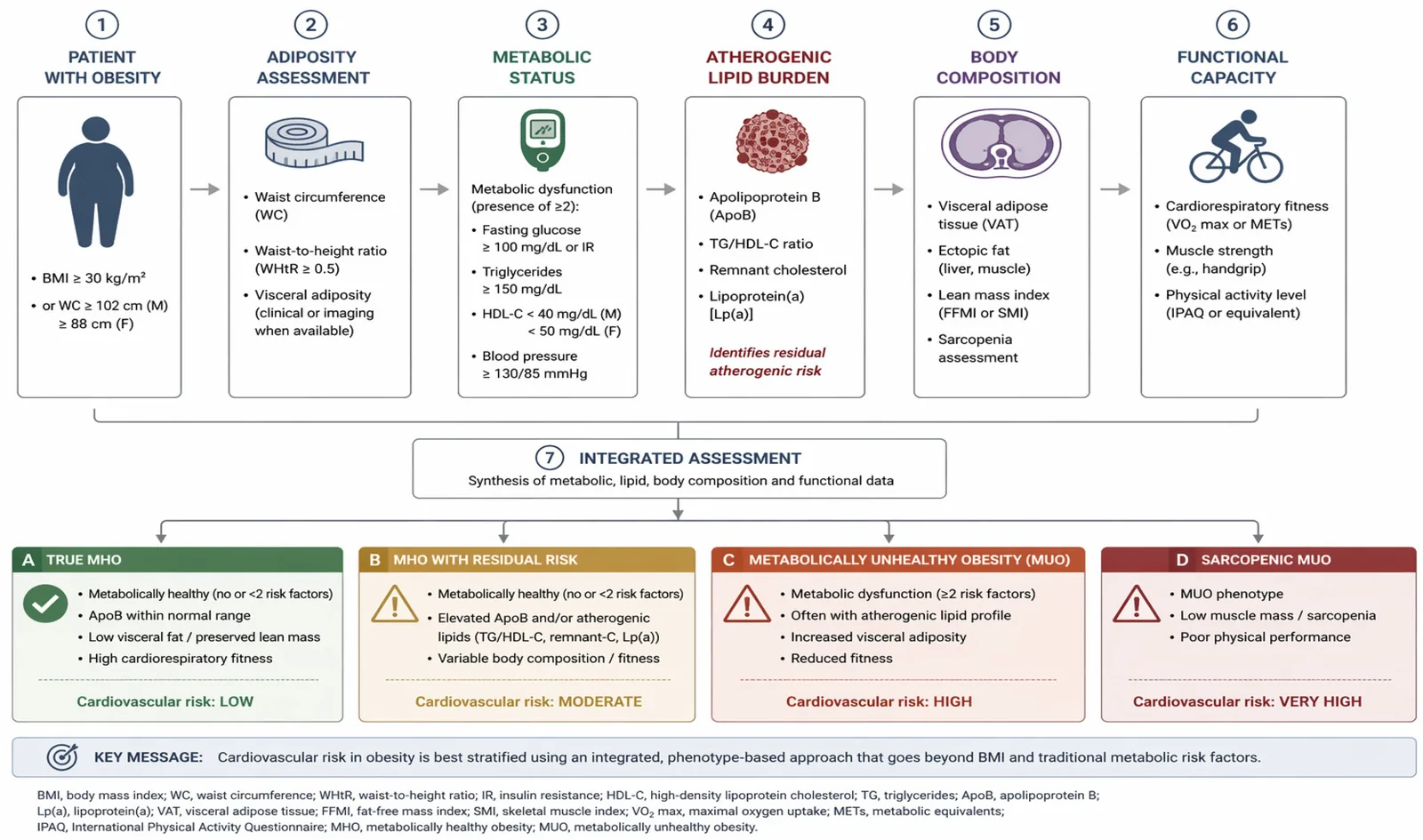
Clinical phenotype-based framework for obesity risk stratification.

Based on the data collected in this review, we propose a stepwise clinical approach integrating adiposity, metabolic status, atherogenic lipid burden, body composition, and functional capacity to help accurately assess CMR in patients with obesity and to determine the type of obesity of an individual, see Figure 3. This model should help distinguish between metabolically MHO, obesity with residual risk, MUHO and/or sarcopenic obesity, enabling improved CVR assessment beyond traditional metrics, but it should be interpreted with caution, as further studies are needed to validate it.

## 6. Discussion

Age and sex substantially influence the metabolic expression of obesity. The prevalence of MHO decreases with advancing age, whereas the likelihood of transition toward MUO increases because of progressive visceral adiposity, skeletal muscle loss, chronic low-grade inflammation, and declining insulin sensitivity. Female sex is generally associated with a higher prevalence of MHO during the reproductive years; however, this advantage diminishes after menopause owing to estrogen deficiency, redistribution of adipose tissue toward the visceral compartment, and worsening CMR. Consequently, age- and sex-specific factors should be incorporated into the clinical characterization of obesity phenotypes [18,20,23,63].

Ethnic background further contributes to the heterogeneity of obesity phenotypes. Asian populations generally develop visceral adiposity, IR, and type 2 diabetes at lower BMI values than White populations, whereas individuals of African ancestry often exhibit lower visceral fat and TG levels despite substantial CMR. These differences indicate that anthropometric thresholds and lipid biomarkers may not perform equally across ethnic groups. Furthermore, many longitudinal cohorts investigating MHO have predominantly enrolled white populations, limiting the generalizability of current evidence to more diverse populations [20,22,24,64].

Future studies should validate phenotype-based classification systems across diverse ethnic populations before universal implementation.

Family history of type 2 diabetes, premature CVD, and obesity may influence the probability of maintaining the MHO phenotype despite similar anthropometric characteristics. Likewise, pre-existing comorbidities, including hypertension, NAFLD, polycystic ovary syndrome, and impaired glucose tolerance, may accelerate the transition toward the metabolically unhealthy phenotype [20,25,26].

Genetic background also contributes to obesity heterogeneity. Variants in genes involved in appetite regulation, adipocyte differentiation, lipid metabolism, and fat distribution modify the probability of maintaining metabolic health despite excess adiposity. Nevertheless, genetic predisposition interacts strongly with environmental and lifestyle factors rather than acting independently [18,24,48].

Lifestyle is consistently recognized as one of the strongest determinants of MHO. Higher levels of physical activity, better cardiorespiratory fitness, adherence to Mediterranean or plant-based dietary patterns, adequate sleep quality, smoking avoidance, and moderate alcohol consumption are consistently associated with maintenance of the MHO phenotype. Conversely, sedentary behavior, poor diet quality, sleep disturbances, and smoking accelerate progression toward MUO, independently of BMI [4,20,39,40].

Medication use should also be considered when characterizing obesity phenotypes. Drugs such as glucocorticoids, atypical antipsychotics, beta blockers, or certain antidiabetic agents may influence body fat distribution, insulin sensitivity, and body composition, thereby modifying the apparent metabolic phenotype [22,54].

The duration and trajectory of obesity are increasingly recognized as clinically relevant determinants. Individuals with long-standing obesity, repeated weight cycling, or progressive weight gain exhibit greater visceral fat accumulation, adipose tissue dysfunction, and metabolic deterioration than those with more recent or stable obesity. Therefore, the duration and trajectory of obesity provide complementary prognostic information beyond a single BMI measurement [6,25,48,65].

Socioeconomic status, educational level, healthcare access, neighborhood characteristics, psychological stress, and food environment further influence obesity phenotypes by affecting diet quality, physical activity, sleep, healthcare, and long-term adherence to healthy behaviors. These factors partly explain the substantial heterogeneity observed among individuals with apparently similar anthropometric characteristics [18,48,61,65].

While the proposed framework integrates multiple complementary markers that may improve CVR stratification in obesity, its implementation in routine clinical practice remains challenging. Advanced biomarkers such as ApoB, imaging-derived measures of visceral adiposity, DXA-based body composition assessment, and cardiorespiratory fitness testing are not universally available and may be limited by cost, infrastructure, technical expertise, and healthcare resources. Therefore, the proposed framework should be regarded as an evidence-informed conceptual model rather than a universally applicable clinical algorithm. Its principal value lies in illustrating how complementary assessments may refine obesity phenotyping, facilitate more individualized CV stratification, and support personalized clinical decision-making when appropriate resources are available [6,7,36].

Moreover, the long-term clinical significance of MHO remains an area of active investigation. Although many longitudinal studies suggest that a substantial proportion of individuals with MHO eventually develop metabolic abnormalities, other cohorts have demonstrated sustained metabolic health over prolonged follow-up. This apparent heterogeneity likely reflects differences in diagnostic criteria, duration of follow-up, body fat distribution, insulin sensitivity, cardiorespiratory fitness, and lifestyle factors. Consequently, MHO should be viewed as a heterogeneous phenotype with variable clinical trajectories rather than a uniformly benign or uniformly transient condition. Recent pharmacological advances further support this concept. Beyond weight reduction, glucagon-like peptide-1 receptor agonists improve visceral adiposity, insulin sensitivity, and several CMR markers, highlighting the importance of targeting adipose tissue quality in addition to body weight [12,18,24,48,61,65].

Obesity should no longer be defined solely by body mass index, but rather by an integrated assessment of fat distribution, lipid burden, skeletal muscle mass and functional capacity [2,21,22]. Incorporating advanced lipid biomarkers and objective measures of fitness enables more accurate identification of residual CVR and supports a shift toward phenotype-based, personalized prevention strategies [37,50].

The terminology of MHO should also be interpreted with caution, as it may inadvertently imply a benign clinical condition. However, accumulating evidence indicates that, despite the absence of overt metabolic abnormalities, some individuals with MHO remain at increased long-term risk of CVD, type 2 diabetes, and all-cause mortality compared with metabolically healthy individuals without obesity. Rather than representing a uniformly low-risk phenotype, MHO appears to encompass a heterogeneous spectrum of CMR, reinforcing the need for multidimensional clinical assessment beyond conventional metabolic criteria. The concept of MHO also shares important similarities with the emerging concept of preclinical obesity, which recognizes that excess adiposity may precede overt metabolic disease and cardiovascular complications [65]. From this perspective, the absence of established metabolic abnormalities should not be interpreted as the absence of biological risk. Rather, both concepts emphasize that obesity exists along a continuum, in which early pathophysiological alterations may already be present despite apparently preserved metabolic health [20,22]. This interpretation further supports longitudinal monitoring and individualized CVR assessment in individuals with obesity [48,65].

In selected individuals with preclinical obesity, pharmacotherapy may also be considered based on the degree of adiposity and the overall risk of progression to obesity-related disease [12].

The existence of such a phenotype has generated intense debate regarding whether a “low-cardiovascular-risk” obesity type truly exists or whether it merely reflects a transient metabolic phase preceding deterioration. Is MHO a true phenotype or a transient state [18,24,66]?

The concept of MHO remains inconsistently defined and clinically controversial. Current classification systems rely predominantly on conventional metabolic criteria, including glucose levels, lipid profile, and blood pressure. While these parameters are essential, they provide an incomplete representation of CVR [18,24,66].

First, traditional definitions fail to account for atherogenic lipoprotein burden, particularly apolipoprotein B and remnant lipoproteins [50,59]. ApoB reveals atherogenic burden not captured by LDL-C, reflecting increased atherogenic particle number and residual CVR that remains undetected by standard lipid panels [50].

Second, these classifications do not adequately capture fat distribution, particularly visceral and ectopic adiposity, which are key determinants of CMR [21,25]. As a result, individuals with normal metabolic markers but high visceral fat may be misclassified as low risk.

Third, current definitions overlook skeletal muscle mass and quality, which are essential determinants of metabolic health [7,44]. Sarcopenic obesity represents a high-risk phenotype that cannot be identified using conventional metabolic criteria alone [44].

Beyond improving aerobic capacity and muscle strength, exercise appears to influence the natural history of obesity phenotypes. Individuals with MHO generally exhibit higher levels of cardiorespiratory fitness than those with MUO, which may partly explain their preserved insulin sensitivity and lower CMR. Regular aerobic and resistance exercise reduces visceral adiposity, improves insulin sensitivity, attenuates chronic low-grade inflammation, and preserves skeletal muscle mass, thereby targeting several mechanisms implicated in the transition from MHO to MUO [17,36,37,38,39,40,61,62].

Longitudinal studies suggest that declines in physical activity and cardiorespiratory fitness are associated with progressive metabolic deterioration, whereas sustained exercise may delay or prevent conversion from the metabolically healthy to the metabolically unhealthy phenotype. Nevertheless, exercise alone does not completely determine phenotype stability, as genetic susceptibility, aging, hormonal changes, dietary habits, and visceral fat accumulation also contribute to metabolic transition [36,37,61,62,65].

These observations support the concept that physical fitness should be viewed not merely as a marker of health status but as a potentially modifiable determinant of MHO maintenance. Exercise interventions may be particularly valuable in preserving the MHO phenotype and delaying progression toward MUO, although long-term randomized studies specifically addressing phenotype transitions remain limited [36,37,38,61,65].

Finally, and perhaps most importantly, CVR is not incorporated into current classification systems, despite growing evidence supporting its independent and powerful association with CV and all-cause mortality [36,37].

Collectively, these observations suggest that current definitions of MHO substantially underestimate CVR and fail to reflect the true biological heterogeneity of obesity. This underscores the need for an integrated, phenotype-based approach [20,21,22].

### Limitations

Unlike systematic reviews and meta-analyses, narrative reviews do not typically involve formal methodological quality assessment, risk-of-bias evaluation, or quantitative synthesis. Consequently, conclusions are based on qualitative critical appraisal of the available evidence and its interpretation. This review has several methodological and conceptual limitations that should be acknowledged.

- First, as a narrative review, this study does not follow the methodological framework of a systematic review or meta-analysis. Although a structured literature search and transparent study selection process were implemented, the narrative design inherently carries a risk of selection bias. Furthermore, no formal methodological quality assessment or risk-of-bias tool (e.g., the Newcastle–Ottawa Scale or ROBINS-I) was applied to the included studies. Consequently, evidence was synthesized through qualitative critical appraisal rather than weighted according to standardized quality scores. To facilitate critical appraisal of the original data, we favored full-text manuscripts, written in English. This approach may have resulted in the exclusion of some relevant, high-quality publications that were not available in full text, free of charge, and could have led to selection biases.
- Second, the proposed integrative framework combining body composition, lipid burden, and functional capacity should be regarded as a conceptual model intended to summarize the current evidence rather than as a validated clinical algorithm. Prospective validation in independent cohorts is required before its implementation in routine clinical practice.
- Third, the lack of a universally accepted definition of MHO, together with substantial heterogeneity in diagnostic criteria, study populations, follow-up duration, obesity classifications, and metabolic assessment methods, limits direct comparisons across studies and may contribute to variability in reported outcomes and the generalizability of the available evidence.
- Fourth, much of the available evidence is derived from observational studies, including cross-sectional and longitudinal cohort studies. Consequently, causal relationships between obesity phenotype, metabolic health, and long-term clinical outcomes cannot be definitively established, and residual confounding by lifestyle, socioeconomic status, genetic predisposition, and medication use cannot be excluded.
- Fifth, metabolic health is a dynamic rather than a static condition. Individuals classified as having MHO may transition to MUO, whereas others may improve their metabolic profile following lifestyle modification or weight reduction. Consequently, single time-point assessments may not accurately reflect long-term CMR.
- Sixth, many studies investigating MHO rely predominantly on conventional metabolic markers (fasting glucose, triglycerides, HDL-C, and blood pressure), which may not adequately capture early metabolic dysfunction. More sensitive indicators, including measures of insulin resistance, ectopic fat accumulation, inflammatory biomarkers, and advanced CVR markers, remain inconsistently assessed across the available literature.
- Seventh, our proposed integrative framework and its associated thresholds lack prospective validation in independent cohorts, which will be the aim of future studies.
- Eighth, differences in ethnicity, sex, age, and geographic background may influence both the prevalence and the clinical significance of MHO. Because specific populations remain underrepresented in the current literature, the generalizability of existing findings may be limited.

## 7. Conclusions

The concept of MHO is associated with lower CVR and CMR than MUHO, but higher risk than metabolically healthy normal weight individuals, and many cases appear transient, for a longer or shorter timeframe. Reliance on conventional metabolic criteria alone obscures significant residual CVR driven by atherogenic lipoproteins, visceral adiposity, and impaired functional capacity. Transitioning from BMI-centered models to an integrated, phenotype-based framework that incorporates body composition, ApoB-related risk, and CRF could help in accurate risk stratification and effective, personalized prevention strategies in obesity.

By bridging pathophysiological mechanisms with practical clinical assessment, this work proposes a clinically applicable strategy for personalized CVR stratification in patients with obesity.

## Figures and Tables

**Table 1:** The most dangerous lipid profile in obesity.

Lipid Parameters	Pathological Threshold/Typical Alteration	Key Mechanism and Clinical Relevance	Current Evidence-Based Management and Emerging Therapeutic Strategies	
			Current Management	Emerging Therapies
TG	≥150 mg/dL (fasting) or elevated non-fasting TG	Hepatic VLDL1 overproduction driven by visceral FFA flux and IR; impaired clearance → residual atherogenicity and endothelial inflammation.	Lifestyle modification Weight loss Mediterrannean diet Exercise Statins (when indicated) Fibrates (selected patients) Icosapent ethyl (selected high-risk patients)	ApoC-III inhibitors ANGPTL3 inhibitors Remnant cholesterol-targeted therapies
HDL-C	<40 mg/dL (men); <50 mg/dL (women)	CETP-mediated TG enrichment and accelerated catabolism; im-paired HDL function (reverse cholesterol transport, antioxidative capacity) contributes to residual ASCVD risk [5,15,57].	Lifestyle Weight loss Exercise Smoking cessation Management of insulin resistance	CETP inhibitors HDL functionality-enhancing therapies;.
LDL-C/particle quality	LDL-C may be normal; shift towards sd-LDL; discordantly high ApoB	CETP exchange + hepatic lipase → sd-LDL: greater arterial entry, oxidation, longer residence time; underestimates risk when LDL-C appears “acceptable” [50].	Lifestyle modification Weight loss Mediterranean diet Regular aerobic and resistance exercise High-intensity statins Ezetimibe PCSK9i Inclisiran	LDL particle-targeted therapies CETP modulators Gene-silencing therapies Precision lipidomic-guided therapy
ApoB	≥80 mg/dL (high-risk) or ≥100 mg/dL (very high atherogenic parti-cle burden)	Direct proxy for number of atherogenic particles (VLDL/IDL/LDL/Lp(a)); captures discordance (normal LDL-C, high particle burden) common in obesity/metabolic syndrome [15,50].	Lifestyle modification Weight loss Statins Ezetimibe PCSK9i Inclisiran	Novel ApoB-targeted therapies ANGPTL3 inhibitors Lp(a)-lowering therapies (pelacarsen, olpasiran) [11,15,47]
TG/HDL-C ratio	>3 (mg/dL units) (surrogate of IR/sd-LDL)	Pragmatic marker of IR and sd-LDL predominance; correlates with NAFLD and CMR clustering [5,18,35,57].	Lifestyle modification Weight loss Mediterranean diet Regular aerobic and resistance exercise GLP-1RA (selected patients) SGLT2i	Insulin sensitivity-targeted therapies Precision metabolic phenotyping Multi-biomarker risk stratification
Remnant cholesterol	Elevated (e.g., ≥30 mg/dL in many cohorts) or increased postprandial remnants	Cholesterol in TG-rich remnants (VLDL, IDL) enters the arterial wall and promotes foam-cell formation and inflammation—causal in atherogenesis [59].	Lifestyle modification Weight loss Mediterranean diet TG TG-lowering therapy Statins Icosapent ethyl (selected patients)	ApoC-III inhibitors ANGPTL3 inhibitors Remnant lipoprotein-targeted therapies
Non-HDL-C	≥130 mg/dL (general high-risk threshold; lower targets in very-high-risk patients)	Composite of all atherogenic cholesterol (VLDL + IDL + LDL+ Lp(a)); aligns with ApoB and additional risk when TG is elevated [11,50].	Lifestyle modification Weight loss Mediterranean diet High-intensity statins Ezetimibe PCSK9i Inclisiran	ApoB-guided lipid lowering Novel ApoB-targeted therapies Gene-silenching therapies [50]

Legend: TG = triglycerides; HDL-C = high-density lipoprotein cholesterol; LDL-C = low-density lipoprotein cholesterol; sd-LDL = small dense low-density lipoprotein; ANGPTL3 = angiopoietin-like protein 3; ApoB = apolipoprotein B; VLDL = very-low-density lipoprotein; IDL = intermediate-density lipoprotein; IR = insulin resistance; CETP = cholesteryl ester transfer protein; FFA = free fatty acids; ASCVD = atherosclerotic cardiovascular disease; GLP-1RA = glucagon-like peptide-1 receptor agonists; SGLT2i = sodium-glucose cotransporter-2 inhibitors; PCSK9i = proprotein convertase subtilisin/kexin type 9 inhibitors [2,26,54]. Treatment recommendations are based on current ESC/EAS dyslipidemia guidelines and complemented by recent evidence on emerging lipid-lowering therapies [60].

## Data Availability

No new data were created or analyzed in this study. Data sharing is not applicable to this article.

## Supplementary Materials

The following supporting information can be downloaded at: https://www.mdpi.com/article/10.3390/jcm15166446/s1, Table S1: Methodological quality assessment of cited narrative reviews using the SANRA tool.

## Abbreviations

The following abbreviations are used in this manuscript:

ANGPTL	Angiopoietin-like protein
ApoB	Apolipoprotein B
ASCVD	Atherosclerotic cardiovascular disease
AT	Anaerobic threshold
BIA	Bioelectrical impedance analysis
BAI	Body adiposity index
BMI	Body mass index
BRI	Body roundness index
CETP	Cholesteryl ester transfer protein
CRF	Cardiorespiratory fitness
CT	Computed tomography
CMR	Cardiometabolic risk
CVR	Cardiovascular risk
CVD	Cardiovascular disease
DXA	Dual-energy X-ray absorptiometry
FFA	Free fatty acids
FFM	Fat-free mass
FFMI	Fat-free mass index
GLP-1RA	Glucagon-like peptide-1 receptor agonists
HDL-C	High-density lipoprotein cholesterol
HIIT	High-intensity interval training
HOMA-IR	Homeostatic model assessment of insulin resistance
HRV	Heart rate variability
IDL	Intermediate-density lipoprotein
IR	Insulin resistance
LAP	Lipid accumulation product
LDL-C	Low-density lipoprotein cholesterol
Lp(a)	Lipoprotein(a)
MET	Metabolic equivalent of task
MRI	Magnetic resonance imaging
MRI-PDFF	Magnetic resonance imaging–proton density fat fraction
MUO	Metabolically unhealthy obesity
MHO	Metabolically healthy obesity
NAFLD	Non-alcoholic fatty liver disease
PCSK9i	Proprotein convertase subtilisin/kexin type 9 inhibitors
RMSSD	Root mean square of successive differences
SAT	Subcutaneous adipose tissue
sd-LDL	Small dense low-density lipoprotein
SMI	Skeletal muscle index
SGLT2i	Sodium-glucose cotransporter-2 inhibitors
TG	Triglycerides
VAI	Visceral adiposity index
VAT	Visceral adipose tissue
VFA	Visceral fat area
VFM	Visceral fat mass
VLDL	Very-low-density lipoprotein
VO_2_max	Maximal oxygen uptake
WC	Waist circumference
WHR	Waist-to-hip ratio
WHtR	Waist-to-height ratio
6MWT	Six-minute walk test
