# Supplementary material for: Insights into Metabolically Healthy Obesity—A Narrative Review
Source: J Clin Med. 2026 Aug 20;15(16):6446. doi: 10.3390/jcm15166446 (PMC13513589; doi:10.3390/jcm15166446)
Supplement: Supplementary file 1 [file jcm-15-06446-s001.zip › jcm-4394000-supplementary.pdf]

Author/Year	Importance	Objectives	Search	Referencing	Reasoning	Data	Score
Thomas et al. (2023)[4]	2	2	2	2	2	2	12
Blaak & Goossens (2023) [20]	2	2	1	2	2	2	11
Piche et al. (2020) [22]	2	2	1	2	2	2	11
Lemieux & Despres (2020) [23]	2	2	1	2	2	2	11
Fahed et al. (2022) [26]	2	2	1	2	2	2	11
Henning (2021)[27]	2	2	1	2	2	2	11
Kurniawan (2024) [32]	2	2	1	2	2	1	10
Prado (2022) [45]	2	2	1	2	2	2	11
Sehayek et al. (2025) [50]	2	2	1	2	2	2	11
Bays et al. (2024) [51]	2	2	1	2	2	2	11
Berberich & Hegele (2022) [54]	2	2	1	2	2	2	11
Kosmas et al. (2023) [57]	2	2	1	2	2	2	11
Koros et al. (2025) [63]	2	2	1	2	2	2	11
Rosero-Revelo et al. (2025) [65]	2	2	1	2	2	2	11

Supplementary Table S1: Methodological quality assessment of cited narrative reviews using the SANRA tool

To further strengthen the methodological rigor of the present review, all cited narrative reviews were independently appraised using the Scale for the Assessment of Narrative Review Articles (SANRA). SANRA evaluates six methodological domains (importance of the topic, statement of objectives, literature search, referencing, scientific reasoning, and presentation of data), with each domain scored from 0 to 2, yielding a maximum score of 12.
